# Operational nursing managers’ experiences of clinical supervision at a Johannesburg Hospital

**DOI:** 10.4102/curationis.v47i1.2521

**Published:** 2024-05-24

**Authors:** Bonginkosi I. Shongwe, Charlene Downing, Sanele Nene

**Affiliations:** 1 Department of Nursing, Faculty of Health Sciences, University of Johannesburg, Johannesburg, South Africa

**Keywords:** clinical supervision, managers, public hospital, mixed method research design, patient safety

## Abstract

**Background:**

Clinical supervision is pivotal in supporting nurses in rendering quality, safe patient care. Therefore, it is essential to understand clinical supervision from operational nursing managers’ context to define existing challenges and propose suitable recommendations.

**Objectives:**

This study aimed to explore and describe operational nursing managers’ experiences of clinical supervision within the context of an academic hospital in Gauteng province and propose evidence-based practice recommendations to improve patient safety and the quality of clinical supervision.

**Method:**

An exploratory, sequential, mixed-method design was used and implemented over two phases to take advantage of the strengths of both the qualitative and quantitative research designs. Unstructured individual interviews were conducted to collect data in phase one, and an adapted Manchester Clinical Supervision Scale (MCSS) questionnaire was used to collect data in phase two.

**Results:**

Operational nursing managers work in stressful conditions and environments with a gross shortage of staff and tools of the trade while being expected to deliver high-quality and safe nursing care. Of the sampled respondents, 36% (*n* = 17) were dissatisfied with the supervision they received, while 64% (*n* = 30) were indifferent in the sense that they did not think it was adequate or inadequate.

**Conclusion:**

Clinical supervisors should be trained and supported in clinical supervision, with regular workshops on interpersonal relations.

**Contribution:**

A clearer understanding of clinical supervision within the hospital context and evidence-based practice recommendations to improve patient safety and the quality of clinical supervision.

## Introduction

Clinical supervision is proposed as a solution-focused approach that supports nurses in effectively managing their work schedules and addressing mounting incidents of missed care in the healthcare environment (Markey et al. 2020:2). Recent media reports indicate a growing concern regarding the quality of care in many hospitals within the Gauteng province, South Africa. Moreover, the authors observed a decline in the quality of nursing care in public hospitals in Gauteng because of poor clinical supervision. This was evidenced by 2974 hospital-acquired pressure injuries reported across Gauteng public hospitals by the clinical audit directorate over the past 3 years (Makua 2018/2019–2019/2020). The increase in patient safety incidents occurred despite public hospitals appointing operational nursing managers to oversee clinical supervision and ensure effective and efficient patient care.

According to the International Council of Nurses, clinical supervision is necessary to improve patient care, develop clinical personnel’s professionalism and maintain ethical standards in the field (Quantum Units Education 2020). Vandette and Gosselin (2019:4) state that all other health professions consider clinical supervision as a professional, empowering and supportive act, offered by an experienced supervisor in the professional field to the supervisee. However, it has been determined that a lack of clear and standardised guidelines on clinical supervision creates uncertainty for operational nursing managers. Furthermore, a lack of clinical supervision standards facilitates a culture of poor clinical supervision practices, resulting in a decline in the quality of nursing care and the development of hospital-acquired pressure injuries. According to the Gauteng Department of Health Strategic Plan (2020 [2024]), the cost of medical litigation for the 2018/2019 financial year amounted to R20 273 909 311, posing a serious financial risk to the Gauteng healthcare system. An analysis of patient-safety incidents confirmed that out of 2974 reported cases, 413 were from the hospital where the study was conducted and 302 of these were caused by poor supervision (Makua 2018/2019–2019/2020). Ning and Costello (2017:2) identified clinical supervision as the best practice to improve quality of care and patient safety. It was therefore imperative to conduct this study as a need exists to explore and describe operational nursing managers’ experiences of clinical supervision within the context of a public hospital in Johannesburg and propose evidence-based practice recommendations to improve clinical supervision.

### Aim and objectives

This article aims to explore and describe operational nursing managers’ experiences of clinical supervision within the context of an academic hospital in Johannesburg, determine operational nursing managers’ level of satisfaction with the supervision they received and its influence on how they supervise and propose evidence-based practice recommendations to improve patient safety and the quality of clinical supervision.

## Research methods and design

### Study design

A mixed-method research design was used in this study, employing a sequential exploratory strategy (Creswell & Creswell 2018:360).

### Phase one: Qualitative phase

In this phase of the study, a qualitative, descriptive phenomenological approach was adopted to explore and describe operational nursing managers’ experiences of clinical supervision in relation to patient safety within the context of an academic hospital in Johannesburg.

#### Setting

The study was conducted at an academic hospital in the Gauteng province, South Africa. The hospital had a 2888-bed capacity divided between five units, namely, medical, surgical, maternity, paediatrics and specialised services.

#### Study population

The population for this study was professional nurses working as operational nursing managers in a permanent or acting capacity within a specific academic hospital in Johannesburg. A sampling frame was created, consisting of 127 professional nurses working as operational nursing managers.

#### Sampling strategy

A purposive sampling method was used in phase one, where the first author purposively selected participants to ensure information-rich cases or cases that could elaborate a great deal on the phenomenon were chosen (Gray, Grove & Sutherland 2017:345). Criteria for selecting participants included those: (1) working as an operational nursing manager; (2) for a period of at least 2 years and (3) with recorded incidences of hospital-acquired pressure injuries in the nursing units they were managing.

#### Data collection

Data were collected using unstructured, individual interviews conducted by the first author at a date, time and venue agreed upon with the participants. Unstructured interviews method was employed because they are loosely formatted, where topics are participant driven, the interviewer might not have a pre-formatted interview guide prior to the interview and talk is more likely to resemble an everyday conversation. Furthermore, Polit and Beck (2018:419) advanced that in qualitative studies, researchers often use unstructured interviews to develop a comprehensive understanding of a phenomenon and provide an opportunity to evaluate the extent to which a consistent and coherent picture of the phenomenon emerges. Nine participants were interviewed before data saturation was reached.

#### Data analysis

The first author transcribed the interviews and used Colaizzi’s descriptive phenomenological method to analyse the data (Morrow, Rodriguez & King 2015:643–644). With this method, data were analysed following seven steps. The first author thus read through all the participants’ accounts, formulated meanings relevant to the phenomenon and clustered identified meanings into themes that were common across all accounts. A full and inclusive description of the findings was written and condensed to produce the fundamental structure, which the first author returned to all participants to ask whether it captured their experiences. No changes were required after descriptions were forwarded to the participants. An independent coder helped to strengthen the dependability of the findings by listening and reading the interview recordings and transcripts and identifying themes and subthemes. The transcripts then discussed with the other authors until consensus was reached on the emerging themes.

### Phase two: Quantitative phase

This was a supplementary follow-up phase. A quantitative method with a comparative descriptive and cross-sectional design was applied to examine changes in a variable over time by comparing its values among several groups in different phases of a process (Gray et al. 2017:207).

#### Setting

Respondents for this phase completed a survey questionnaire within the hospital, in a place free of intimidation and prejudice, chosen by the respondent. The accessible population met the specified inclusion criteria and consented to participate in the study.

#### Study population

Respondents were recruited from operational nursing managers who met the inclusion criteria stipulated for phase one and included those who were interviewed. The sampling frame comprised a list of 127 professional nurses working as operational nursing managers in the hospital.

#### Sampling strategy

A stratified random sampling method was used to recruit respondents into the study, and the sample size was 50% of the sample drawn from each stratum. The variables used for stratification included the classification of the nursing units where hospital-acquired pressure injuries occurred, nursing management-related work experience, age, gender and level of qualification.

#### Data collection

For this phase, data were collected using the Manchester Clinical Supervision Scale (MCSS) questionnaire, in which, 68 responses were received. The tool was adapted by the first author to include the measure of the role played by age, post-basic qualifications in nursing management, nursing management work experience in clinical supervision, the extent of satisfaction that operational nursing managers had with the supervision they received and proposals for clinical supervision improvement strategies.

#### Data analysis

The quantitative data analysis process involved data preparation and statistical analysis. In the preparatory phase, data were captured, cleaned and then coded (Grove & Gray 2019:378). The first author captured the data from hard copies to electronic form. The data were then submitted to the statistician for an accurate review of the entries. The numerical data were then computed for analysis by a statistician from STATKON (University of Johannesburg) using the International Business Machines Corporation (IBM) Statistical Package for the Social Sciences (SPSS), version 26. The analysis procedures included descriptive statistics to summarise the data; factor analysis to reduce the data; correlation analysis to identify and explain relationships in the data; comparative analysis to identify and explain significant differences in the data and path analysis to identify and describe the quality of clinical supervision factors that exerted the greatest influence on patient safety (Grove & Gray 2019:378).

#### Reliability and validity

The reliability coefficient for the total scale was 0.860, and the intra-class correlation coefficient for test-retest reliability for the total scale, comprised the seven subscales, was above 0.93 (Cruz 2011:51–56). Three experts tested the MCSS’s face validity in clinical supervision in nursing. They were asked to assess the content of the scale and examine the words and sentences to confirm they were easily understood. Construct validity was tested by examining the items’ internal consistency (Cruz 2011:51–56).

### Ethical considerations

Ethical clearance to conduct the study was obtained from the Research Ethics Committee (REC-823-2020) and the Higher Degrees Committee of the Faculty of Health Sciences at the University of Johannesburg. Permission to conduct the study was also obtained from the Gauteng Department of Health Policy, Planning and Research Directorate.

## Results

### Phase one participants’ demographics

Nine participants were selected using a purposive sampling method to obtain information-rich cases or cases that could teach the authors much about the phenomenon under study (Gray et al. 2017:345). The sample comprises primarily black female nurses with varying qualifications and experience levels. The age range of the nurse’s spans from 40 years to 60+ years, with the majority falling within the 40 years – 50 years and 50 years – 60 years age brackets. Qualifications include a Bachelor of Nursing Education and Administration (BCur Ed & Admin) with specialisations in occupational health, advanced midwifery, critical care nursing and child nursing care. Additionally, there are individuals with Diplomas in Nursing Administration, Orthopaedic Nursing, Child Nursing and Nursing R 425. Experience levels range from 2 to 8 years, indicating a mix of both seasoned professionals and those with relatively fewer years in the field. Four participants had less than 5 years of experience as operational nursing managers, but they had a post-basic qualification in nursing management. The participants were from all five functional business units and shared complex yet similar experiences with clinical supervision and patient safety. Despite having different years of experience as operational nursing managers and qualifications in nursing management, they were all dissatisfied with the quality of clinical supervision they received in their units and had varying views on patient safety. Table 1 presents a summary of the participants’ biographic data.

**TABLE 1:** A summary of participants’ biographic data.

Participant	Age (years)	Gender	Highest qualification	Ethnicity	Years of experience
Participant 1	40–50	Female	BCur Ed and Admin and Occupational Health	Black person	8
Participant 2	60+	Female	BCur Ed and Admin and Advanced Midwifery	Black person	6
Participant 3	40+	Male	Diploma Nursing Admin and Orthopaedic Nursing	Black person	3
Participant 4	40–50	Female	BCur Education and Admin and Critical Care Nursing	Black person	2
Participant 5	50–60	Female	Diploma in Child Nursing and BCur Education and Administration	Black person	5
Participant 6	50–60	Female	Diploma in Nursing R 425	Black person	2
Participant 7	50–60	Female	BCur Education and Admin and Child Nursing Care	Black person	6
Participant 8	40–50	Female	Diploma in Nursing Administration	Black person	2
Participant 9	50–60	Female	Diploma in Nursing	Black person	8

*Source:* Shongwe, B.I., 2023, ‘The quality of clinical supervision and patient safety: operational nursing managers’ experiences in a Johannesburg hospital’, Masters thesis, Department of Professional Nursing Practice, University of Johannesburg

### Emergent themes

Three major themes and several categories emerged from one open-ended question: *‘What is your experience of clinical supervision within the nursing units for which you are responsible?’* These themes are described in Table 2.

**TABLE 2:** Research findings – Themes and categories.

Themes	Categories
1. Participants experienced dysfunctional management systems and deteriorating patent safety, fuelled by a ‘check-on’ instead of a ‘check-in’ relationship that lacks appropriate social support between operational nursing managers and their supervisors	1.1 Participants experienced inappropriate supervision, a lack of emotional support and instead felt bullied and scared 1.2 Participants experienced unstructured development with insufficient orientation and induction 1.3 Participants experienced the absence of listening, with insufficient and inadequate physical support 1.4 The participants’ positions demanded various immense administration responsibilities
2. Participants experienced limited job satisfaction and endured profound frustration with insufficient resources to do their work properly	2.1 Participants’ job satisfaction was greatly compromised because of a lack of essential resources and a shortage of staff 2.2 Participants mentioned a lack of sufficient and well-functioning equipment
3. Participants expressed their essential needs in this position and the need for a structured clinical supervision process, which will also improve the quality of nursing care	3.1 In order to deliver a good service as an operational manager, they expressed their need for: structured mentorship regular visits from their supervisors urgent resources

*Source:* Shongwe, B.I., 2023, ‘The quality of clinical supervision and patient safety: operational nursing managers’ experiences in a Johannesburg hospital’, Masters thesis, Department of Professional Nursing Practice, University of Johannesburg

#### Theme 1: Participants experienced dysfunctional management systems and deteriorating patient safety, fuelled by a ‘check-on’ instead of a ‘check-in’ relationship that lacks appropriate social support between operational nursing managers and their supervisors

The authors’ prolonged engagement with the participants’ narratives revealed that operational nursing managers perceived clinical supervision as a process of finding mistakes instead of a monitoring and supportive exercise. This perception was fuelled by unstructured, unsupportive and unhealthy relations between operational nursing managers and their supervisors, which left them feeling helpless and fending for themselves.

*Category 1.1: Participants experienced inappropriate supervision, a lack of emotional support and instead felt bullied and scared*: Participants revealed that they perceived clinical supervisors (CSs) maintained a ‘check-on’ relationship with them instead of a ‘check-in’ relationship. They had seldom been asked what they needed and how they could be supported. The lack of clear expectations also led them to feel lost and left on their own. The CSs’ visits mainly focused on conducting inspections rather than what really matters, and they did not listen to the participants. Some participants experienced that the CSs showed improved interest, but they mostly felt alone, struggling with minimum resources while attempting to improve the quality of care. The nature of the relationship was not one of good rapport and not one that contributed to professional or personal growth, as demonstrated in the following quote:

‘[*A*]t work I expect that my supervisor, as the first thing, to tell me what is expected and what she expects from me, you understand … Secondly, after telling me that she must give me equipment so that she is able to gauge how I work … Thirdly, she is supposed to support me towards my journey, to say okay am I doing it right or is there where I am lacking, she must support me, not harass me in my job.’ (Participant 2, Female, 60+ years)

Holland, Cooper and Sheehan (2017:925–926) argue that supervisors’ support, having a direct voice, and trust are positively associated with employee engagement. The latter creates a positive work-related psychological and motivational state that reflects a genuine willingness among employees to invest effort in their work role to achieve organisational goals and success. According to Mabona, Van Rooyen and Ten Ham-Baloyi (2022:3), creating an empowering work environment through evidence-based transformational leadership is a practice that depends on a relationship built on trust and respect. Abusive supervision has adverse effects on the subordinate’s physical and mental behaviours, leading to an overall negative impact on the organisation (Wang et al. 2019:152).

*Category 1.2: Participants experienced unstructured development with insufficient orientation and induction*: The participants missed out on proper mentorship during the first few months in their positions. Most participants felt the 2 weeks’ induction they received when starting in the post was too short, and induction topics were not relevant to their daily tasks. The lack of a structured teaching and support process left the participants to ‘run with it’ and ‘shoot from the top’. One participant expressed that this situation made her feel hijacked. Participants also felt overwhelmed and lost because of the sudden scope of responsibility their positions required and the lack of a proper induction process. Participants wanted to learn and understand their positions but experienced that the CSs did not take enough time to train them to use the systems and processes required for their jobs. Clinical supervision was mostly casual, and patient safety was thus compromised. The following participant’s statement highlights these assertions:

‘The first day I start … I became an Operational Manager I was just put in a ward on that Monday morning. Let’s say we did like an orientation for a ‘weeknyana’ those few days and then from there in the morning the following week we are put in a … in a ward and then we had to run the ward …’ (Participant 4, Female, 40 years – 50 years)

Mchete and Shayo (2020:185) postulate that new employees who join organisations come with varied expectations, some of which can be unrealistic. They argue that an intensive orientation programme conducted by professional individuals in the organisation is a vital tool to remove such unrealistic expectations. According to Priya, Venkatesan and Sonia (2018:2), Lalithabai et al. (2021:188), the goal of the nursing induction programme is to ensure newly joined and new graduate nurses receive consistent information regarding policies, procedures, standards and documentation to support practice and help staff become familiar with them.

*Category 1.3: Participants experienced the absence of listening, with insufficient and inadequate physical support*: The participants shared that they did not have the ‘backup’ of their CSs as there is no open-door policy; they had to make an appointment before they could consult with them. Some did not see their CSs often, while others said their CSs visited them daily. While the physical presence of the CSs could have been a positive experience for the participants as a sign of support, the CSs seemed to focus on whether the work was performed correctly, not assisting the participants. Participants also shared that they sometimes felt bullied and scared because of the negative attitude CSs displayed. The following quotes from an operational nursing manager’s narrative illustrate these observations:

‘As a supervisor you are supposed to listen to your people of course, because you show them that when it is tough the people are going to tell you something, but if you don’t give them an opportunity how …’ (Participant 7, Female, 50 years – 60 years)

Another participant further added that:

‘[*A*]rea manager they just come from nowhere and they come and demand things! That you were never told of.’ (Participant 4, Female, 40 years – 50 years)

According to Mabona et al. (2022), it is essential for nurses to take part in decision-making processes involving patient care, and they should also have significant professional autonomy and control over their surroundings. This view is further supported by Kester and Wei (2018:45), who posit that engaging staff in the planning, rollout and maintenance of interventions can provide a sense of team spirit, teamwork, trust and ownership, which, in turn, can positively influence the work environment.

*Category 1.4: The participants’ positions demanded various immense administration responsibilities:* Operational nursing managers expressed feeling overwhelmed by the administrative demands of their posts. The situation was exacerbated by the insufficient support they received from their supervisors. Some responsibilities involved learning new processes for ordering and controlling stock; writing incident reports (which they mostly do not get to because of all their tasks); updating performance management and development system documents, which takes a lot of time; auditing patient records; administrative tasks and keeping a record of all interventions with patients and supervising staff in all aspects of nursing care. The following is one of the participants’ statements:

‘I have so much, so am I ordering or not, so if the other one is in room 7 in the toilet, the other one is in room 3 in the toilet what’s going to happen … but I don’t have a choice … because most of the time now we are busy with the PMDS I will sit here for the entire day not even writing the off duties busy with the PMDS, they are just doing their own things there, patients oh my God are dying which are not supposed to die.’ (Participant 4, Female, 40 years – 50 years)

Medzo-M’engone (2021:344) concluded that job demands have a negative impact on employees or are negatively associated with their psychological well-being. This view is further supported by Bakker and De Vries (2021:16), who argued when employees are confronted with increased job strain, they are more likely to use maladaptive self-regulation strategies such as avoidance and self-undermining. Advanced organisational resources, such as human resource practices and healthy leadership, may help employees to regulate their short-term fatigue.

#### Theme 2: Participants experienced limited job satisfaction and endured profound frustration with insufficient resources to do their work properly

Participants’ feedback about the availability of resources shows a generalised lack of staff and equipment, which affects the quality of care and patient safety, and contributes to low job satisfaction among nursing workforces. Participants also shared that they sometimes take money from their own pockets to generate work-related paperwork, such as printing, as the hospital does not have a functional printer for them to use. They expressed that available nurses had to perform double the work because of staff shortages, which led to burnout and poor work experiences.

*Category 2.1: Participants’ job satisfaction was greatly compromised because of a lack of essential resources and a shortage of staff*: Participants expressed that staff who resigned were not replaced, increasing the workload for those left behind. In addition, absenteeism posed a serious challenge to maintaining consistent patient care. Agency staff (moonlighters) had to be used, and participants experienced that their CSs did not understand the need for sufficient staff to maintain high-quality patient care. Some participants expressed frustration with the competency and quality of nurses allocated in their wards. They indicated that some were incapable of providing safe patient care and required strict, direct supervision, which added an additional burden on them. A participant explained:

‘For starters … eish Mr. What I can say Patient Safety requires human resources and we don’t have enough personnel neh, ama nurses they are short staffed ngempela [*for real*].’ (Participant 7, Female, 50 years – 60 years)

According to Senek et al. (2020:5), nursing managers experience frustration and hopelessness while replacing rota gaps with temporary agency staff because the temporary nurses are unfamiliar with the ward and patients. Navajas-Romero, Ariza-Montes and Hernández-Perlines (2020:10) also postulate that managers should promote a cooperative work environment based on an organisational culture of support, and teamwork should be encouraged.

*Category 2.2: Participants mentioned a lack of sufficient and well-functioning equipment*: The participants struggled with a severe lack of sufficient and functioning equipment. They shared that they had to plead and beg for months for something as minor as a printer and then received one used by other departments. A shortage of or malfunctioning equipment and the lack of a smooth process to order and receive the needed equipment left them feeling unsupported and powerless, as well as frustrated. A participant shared:

‘[*W*]hen you run short of anything, of staffing mostly but if you run of stock in the … in your … in your ward and then you … we literally go to stores to collect for ourselves. That’s what we do. … “Please, can you … go to the stores and go fetch paper towels for us. We don’t have paper towels, we don’t have this, this, this, this” and then we go and collect …’ (Participant 3, Male, 40+ years)

A lack of staff, equipment and needed supplies to care for patients are dissatisfying factors that negatively influence individuals’ quality of work life (Akter, Akter & Turale 2019:39). According to Moyimane, Matlala and Kekana (2017:6), a shortage of medical equipment has a negative impact on patients, the hospital and nursing profession; as such, it creates a barrier to the health system’s functioning. As a result of the unavailability of resources, operational nursing managers experienced poor job satisfaction and felt neglected.

#### Theme 3: Participants expressed their essential needs in this position and the need for a structured clinical supervision process, which will also improve the quality of nursing care

Participants expressed the need for a structured clinical supervision process, where supervisors spend more time in the units, providing guidance and support. Participants further expressed a need for policies and guidelines to revert to when supervisors are unavailable. The participants mentioned the need for supervisors to offer balanced supervision that focus not only on coming to the unit when there is a patient-safety incident but also on the quality of care.

*Category 3.1: In order to deliver a good service as an operational manager, they expressed their need for structured mentorship, regular visits from their supervisors and urgent resources:* Participants wanted someone to hold their hand during the first few months through structured mentorship. They also required regular visits from their supervisors not only in the context of an inspection but also to be asked if they needed help or staff. Participants wanted to be taken seriously if they needed urgent resources and mentioned the need for a professional relationship with the CSs based on trust. They need their CSs to share their professional knowledge of clinical supervision and wanted sufficient opportunities to develop nurses (to teach and to be taught) who love nursing. The following statement from a participant illustrates these observations:

‘I think it’s … they … they’ll come and check when already you’ve done it and then they’ll tell you “No, correct this. This is how it’s done; this is the way it’s supposed to be done” but then you don’t … you just figure out yourself … That’s how I did it myself …’ (Participant 8, Female, 40 years – 50 years)

The participants’ views are shared by Broetje, Jenny and Bauer (2020:15), who claim the key resources operational nursing managers require are supervisor support, fair and authentic management, transformational leadership, interpersonal relations, autonomy and professional resources. Navajas-Romero et al. (2020:10) concur that high job demands, low job control and low social support are negatively correlated. Esteves et al. (2019:2) define ‘support’ within the context of clinical supervision as a process of increasing the quality of professional practice, supporting work and learning that helps professionals develop knowledge and skills. It entails the ability to take responsibility for their actions and improve patients’ safety in complex situations.

## Discussion

### Phase one: Qualitative phase

Operational nursing managers’ lived experiences of the quality of clinical supervision and patient safety, as narrated during participant interviews, revealed they perceive clinical supervision as a process of finding mistakes instead of a monitoring and supportive exercise. It was clear from all the interviews that the participants experienced an immense lack of support in their responsibility-laden positions. The participants seldom been asked what they needed and how they could be supported, and a lack of clear expectations led them to feeling lost and left on their own. They experienced limited job satisfaction and endured profound frustration with insufficient resources to do their work properly. Most participants felt the 2 weeks’ induction they received when starting in the post was too short, and most topics were irrelevant to their daily tasks.

The lack of a structured teaching and support process meant participants had to just ‘run with it’ and ‘shoot from the top’. Clinical supervision was mostly casual, and patient safety was compromised. Participants sometimes felt bullied and scared because of the negative attitude CSs displayed. They developed peer support groups on WhatsApp as a coping mechanism in order to survive as an operational nursing manager. Participants also expressed the need for someone to hold their hand during the first few months in the form of structured mentorship and regular visits from their supervisors not only in the context of an inspection but also to be asked if they need help or staff and to be taken seriously if they do need urgent resources. They emphasised the importance of a professional relationship with the CS based on trust.

### Phase two: Quantitative phase

A majority of respondents (87%; *n* = 41) were females, and 13% (*n* = 6) were males. This was expected as the hospital’s operational nursing manager population was 69% females. The hospital had a high population of operational nursing managers (48.9%; *n* = 23) aged between 51 and 60 years, and 12.8% (*n* = 6) of the respondents were between the ages of 30 and 40. This demonstrated that although the hospital had numerous older operational nursing managers, it was also introducing young people into the operational nursing manager category. Regarding nursing management work experience, it was determined that most respondents (42.6%; *n* = 20) had less than 3 year’s nursing management-related work experience, while 34% (*n* = 16) had 10 years and longer experience in management. This reflects unbalanced succession planning, as most operational nursing managers had less than 3 years’ experience in their position. It was also determined that 74% (*n* = 35) of the respondents had a qualification in nursing management (Table 3).

**TABLE 3:** Nursing management-related work experience.

Years of experience	Frequency	%	Valid %	Cumulative %
**Valid**				
0–3	20	42.6	42.6	42.6
4–6	6	12.8	12.8	55.3
7–9	5	10.6	10.6	66.0
10 or longer	16	34.0	34.0	100.0

**Total**	**47**	**100.0**	**100.0**	**-**

*Source:* Shongwe, B., 2023, ‘The quality of clinician supervision and patient safety: Operational nursing managers experiences in a Johannesburg Hospital’, unpublished Masters, University of Johannesburg, Johannesburg

In relation to the trust and rapport subscale, most respondents (76.5%; *n* = 36) concurred that clinical supervision made operational nursing managers more aware of areas of skill they need to improve (item 6, mean [*M*] = 3.74, standard deviation [s.d.] = 1.073), while 19.1% expressed that supervisors were never available when needed. Respondents rated ‘learning from my supervisor’s experience’ (item 2, *M* = 3.26, s.d. = 1.224) low. A moderate number of respondents also agreed that ‘without clinical supervision, the quality of client/patient care would deteriorate’ (item 3, *M* = 4.09, s.d. = 0.855), and they strongly disagreed that ‘it is important to make time for clinical supervision sessions’ (item 4, *M* = 4.28, s.d. = 0.949). Regarding the extent of satisfaction operational nursing managers have with the supervision they received and its influence, 36% (*n* = 17) of the respondents were dissatisfied with the supervision they received, while 64% (*n* = 30) were indifferent, in that they did not think it was adequate nor inadequate. Moreover, 66% (*n* = 31) reported that they think the way they are supervised influences the way they supervise. In relation to patient safety, 38.3% (*n* = 18) of the respondents viewed patient safety as worse in 2019/2020 compared to 2018/2019, and they recommended that continuous clinical supervision is important to improve patients’ experiences of care and reduce patient-safety incidents and litigations.

### Overall discussion

Creating an empowering work environment through evidence-based transformational leadership is one of the leadership practices that depend on a relationship built on trust and respect (Mabona et al. 2022:3). Abusive supervision has adverse effects on subordinates’ physical and mental behaviours, leading to an overall negative impact on the organisation (Wang et al. 2019:152). The lack of trust and a positive practice environment contributes towards a lack of self-determination and inability to take decisions on the operational nursing managers. Autonomy in decision-making and flexibility is essential in nursing management, as employees are proud of being given freedom in decision-making and setting their own goals without interference from senior management (Lee 2021:107–108).

According to Priya et al. (2018:2) and Lalithabai et al. (2021:188), the goal of the nursing induction programme is to ensure newly joined and new graduate nurses receive consistent information regarding policies, procedures, standards and documentation to support practice and become familiar with them. The objective is to ensure existing resources are used to meet the new demands of a constantly changing healthcare environment. According to Cho, Sagherian and Steege (2021:2), organisational resources, when readily available, help workers cope with work and life stressors and maintain healthy work environments. The participants’ views are shared by Broetje et al. (2020:15), who advance that the key resources operational nursing managers require are supervisor support, fair and authentic management, transformational leadership, interpersonal relations, autonomy and professional resources. It could be concluded that the evidence supports other research findings in the sense that an unsupportive and disorganised work environment contributes to poor clinical supervision, which in turn contributes to poor clinical outcomes and patient safety incidents.

### Strengths and limitations

This study was limited to operational nursing managers employed within the academic hospital in Johannesburg. It is observed that male participants were underrepresented in the qualitative phase, because of the unavailability of some of the male operational nursing managers on the days that interviews were scheduled. However, although the findings on gender in this study do not reflect that of the general population, they are justifiable based on the setting where the study was conducted.

### Implications or recommendations

#### Recommendations for nursing education

Nursing education and training institutions should prioritise the inclusion of a clinical supervision module in the nursing management curriculum, with the aim of enhancing patient safety. Recommendations for nursing education that surfaced from this study include:

- A comprehensive competency-based clinical supervision framework should be developed and implemented to inform a structured programme.
- Nursing education and training institutions should include clinical supervision at both the undergraduate and postgraduate levels.
- Workshops on clinical supervision should be conducted, targeting nursing managers at all levels.

#### Recommendations for nursing research

The study’s findings unveiled significant and valuable insight regarding clinical supervision and patient safety based on operational nursing managers’ perspectives at the academic hospital in Johannesburg. It is recommended that:

- A study could be conducted to explore assistant nursing managers’ experiences with clinical supervision.
- A follow-up study could be conducted to assess whether the identified recommendations were implemented.
- The recommended interventions’ success in improving the quality of clinical supervision should be explored.

#### Recommendations for nursing policy development

The study’s findings can be used in policy development. Recommendations are as follows:

- Develop and implement a clinical supervision policy to guide nursing managers in the clinical supervision process.
- The policy should include strategies to guide assistant nursing managers in creating an enabling environment to assist operational nursing managers during clinical supervision.
- The policy should include mechanisms for the nursing service manager to monitor adherence to the policy.

## Conclusion

The findings underscore the crucial role of evidence-based transformational leadership in fostering an empowering work environment for nursing staff. In addition, it highlights the significance of providing adequate organisational resources and support to operational nursing managers to ensure optimal clinicºal supervision and ultimately enhance patient safety and clinical outcomes. Based on the study’s findings, it has become evident that quality clinical supervision is critical to ensure patient safety. If a quality clinical supervision programme can be developed and implemented, it will likely improve patient safety.
